# ALSPAC Mercury Study and Fish Consumers

**DOI:** 10.1289/ehp.1307757

**Published:** 2014-02-01

**Authors:** Michael Gochfeld, Joanna Burger, Alan H. Stern, Gary Ginsberg, Henry Anderson

**Affiliations:** 1 Rutgers University Environmental and Occupational Health Sciences Institute, Piscataway, New Jersey, USA; 2 Rutgers University School of Public Health, Piscataway, New Jersey, USA; 3 Connecticut Department of Health, Hartford, Connecticut, USA; 4 Wisconsin Division of Public Health, Madison, Wisconsin, USA

Golding et al. (2013) described regression analysis of dietary contributions to maternal blood mercury levels, nested within the Avon Longitudinal Study of Parents and Children (ALSPAC). Fish intake explained only about 7% of the variance in blood mercury, leading them to conclude somewhat cautiously that “limiting seafood intake during pregnancy may have a limited impact on prenatal blood mercury levels” (Golding et al. 2013). The media, however, has been quick to overinterpret the results, and Golding herself was quoted:

We were pleasantly surprised to find that fish contributes such a small amount … to blood mercury levels…. We hope many more women will now consider eating more fish during pregnancy. (ALSPAC 2013)

This is a much less cautious conclusion.

ALSPAC and Golding are responsible for many valuable publications on human development, but ALSPAC was not designed to investigate mercury exposure and effects. The categories of white fish, oily fish, and shellfish used by Golding et al. (2013) do not meaningfully reflect mercury content. With respect to the internal validity of the exposure estimate from the dietary questionnaire, high-mercury and low-mercury fish are represented among both the “white” and “oily” categories, and shellfish generally have very little mercury. Thus, internal validity is limited by a poor exposure metric.

External validity is more of a problem. The results of Golding et al. (2013) are not generalizable to the frequent consumers of fish who are most vulnerable to methylmercury exposure from fish during pregnancy. Women who ate fish frequently (> 3 times/week) made up < 2% of the ALSPAC subsample, and 60% and 76% consumed white fish or oily fish, respectively, no more than every 2 weeks (19% and 42% never consumed either). Moreover, < 1% had a blood mercury concentration > 5.8 µg/L, the level corresponding to the U.S. Environmental Protection Agency (EPA) methylmercury reference dose of 0.1 µg/kg/day (U.S. EPA 2013).

Although frequent fish consumers are a small part of the general population and the ALSPAC study, they are the population at risk for methylmercury exposure, particularly during pregnancy. Frequent fish consumers should reduce frequency or size of fish meals or choose among the many types of fish low in mercury.

The mercury in fish is predominantly methylmercury, and for most people, fish is the only significant source of methylmercury (Björnberg et al. 2005; Mahaffey et al. 2004). Methylmercury is the toxic form that is almost 100% absorbed from the gut, and it is readily translocated to both the brain and the fetus. This is the reason for public health concern regarding consumption of high-mercury fish by frequent fish-eaters. At low levels of fish intake, other, nonorganic forms and pathways influence blood mercury levels.

The results of Golding et al. (2013) suggest that women who rarely eat fish and already have a low blood mercury concentration will not further lower the mercury by eating even less fish. The study, however, did not address the risk for the frequent fish-eater who has elevated blood mercury.

It is not clear whether the developmental benefits ascribed to eating fish are due to its nutrients or to the healthy lifestyles that often correlate with eating fish frequently.

We conclude that women who eat fish rarely or never may benefit both self and fetus by eating fish occasionally. Pregnant women should choose low-mercury fish species, particularly if they eat fish frequently (more than twice a week).
